# Interdisciplinary workshop in the philosophy of medicine: medical knowledge, medical duties

**DOI:** 10.1111/jep.12237

**Published:** 2014-12-02

**Authors:** Emma Bullock, Elselijn Kingma

**Affiliations:** ^1^ Department of PhilosophyCentral European UniversityBudapestHungary; ^2^ School of HumanitiesUniversity of SouthamptonSouthamptonUK; ^3^ Technical University EindhovenEindhovenThe Netherlands

**Keywords:** communication, duty of care, evidence‐based medicine, Hippocratic Oath, interdisciplinarity, medical particularism, medicine, person‐centred medicine, philosophy, public goods, workshop

## Abstract

On 27 September 2013, the Centre for the Humanities and Health (CHH) at King's College London hosted a 1‐day workshop on ‘Medical knowledge, Medical Duties’. This workshop was the fifth in a series of five workshops whose aim is to provide a new model for high‐quality, open interdisciplinary engagement between medical professionals and philosophers. This report identifies the key points of discussion raised throughout the day and the methodology employed.

## Introduction

On 27 September 2013, the Centre for the Humanities and Health (CHH) at King's College London^1^ hosted a 1‐day workshop on ‘Medical Knowledge, Medical Duties’. This workshop was the fifth in a series of five workshops whose aim is to provide a new model for high‐quality, open interdisciplinary engagement between medical professionals and philosophers. Previous workshops focused on concepts of health and disease [1], personhood and identity in medicine [2], death [3] and bodies and minds in medicine [4].

The workshop used the methodology developed over the course of the workshop series, adopting the following six characteristics: (1) more time devoted to plenary discussion than to introductory speakers; (2) matched multidisciplinary introductions providing two points of view on each topic, with the philosopher following on from, and commenting on, the health care professional; (3) equal participation of all participants in a plenary chaired discussion – that is, not merely a question/answer session – facilitated by preparatory reading and a rotating chair; (4) a physical roundtable format and strict limits on the numbers of participants; (5) a diverse and balanced group of participants with strong continuity among participants in the different workshops of the series; and (6) the use of the following discussion conventions: the ‘Canberra rules’ (a method for differentiating comments that introduce a new topic for discussion and comments that are on an existing line of discussion) and ‘pink jargon/clarification card’ (signalling the use of disciplinary jargon in need of clarification). More details on this methodology and why/how we arrived at it are reported here [1, 2]. We consider this methodology crucial to the success of our workshops, and believe that it could be usefully employed in other settings.

Over 40 participants attended the workshop (20 women and 21 men) with around half having attended previous workshops. The demographic of the workshop matched that in previous years: roughly one‐third of the participants came from outside of the UK and the group as a whole was evenly divided between clinical and humanities backgrounds. Background reading material was distributed to the attendees before the event in order to facilitate discussion [5, 6, 7, 8, 9, 10, 11].

## Session 1: public and private goods

The first session was opened by Professor Trisha Greenhalgh (Professor of Primary Health Care and Dean for Research Impact, Barts and the London School of Medicine and Dentistry). Greenhalgh defined public goods as goods that are *non‐rival* and *non‐excludable*. Non‐rival means that my use of a good does not diminish your ability to use it, and non‐excludable means that we cannot in practice stop people from partaking in it. A classic example is clean air; my use of clean air does not diminish your use of it, and if we have clean air, we cannot exclude individuals from benefitting from it [12]. Public health and health care, Greenhalgh suggested, have many characteristics of being a public good, and this provides a useful framework to understand certain problems.

Often, Greenhalgh contended, health care administrators and users experience a conflict between doing what is in their private best interest and doing what contributes to the public good. This raises philosophical and ethical difficulties with protecting public goods. Greenhalgh gave several examples.

First, during a chickenpox outbreak, Greenhalgh's general practitioner (GP) practice employed two separate waiting rooms as a means of limiting contagion and protecting public health: one for patients with (suspected) chickenpox and one for patients without. Patients with unconfirmed cases of chickenpox, however, were reluctant to wait in the former. For while it was in the public interest that they wait in that room, it was against their private interest; since they might not have the virus, waiting in that room would increase their exposure risk.

Another example focussed on the introduction of the ‘summary care record’. This is a national programme to put a summary of patients' medical details on a central database, which would be particularly useful for quick access during emergency and out‐of hour's care. Greenhalgh argued that GP's did not cooperate with the introduction of this programme because the uploading and reorganization of their own data was burdensome, costly and time‐consuming – and thus against their private interest. Those very same GPs, however, felt that the summary care record would be of great use to them in out‐of‐hour's surgery. This, Greenhalgh said, is a classic example of a ‘free rider’ problem. Because public goods, such as the summary database, are non‐excludable, agents will benefit from it whether they contribute to the maintenance of the public good or not. Where this individual contribution is individually costly, therefore, agents do not have much incentive to make that contribution.

In the final example, Greenhalgh focused on the nature of global public goods in relation to global communicable disease control. Greenhalgh noted that because of a lack of access to health care, an increase in male human trafficking across southern African countries has led to a huge increase in tuberculosis and HIV in the population. Greenhalgh suggested that the solution to this public health crisis would require a collaborative effort between the affected countries to provide health care to its populations. This solution, however, raises the possibility of entire countries acting as ‘free riders’; populations may benefit from access to health care in other countries even if their country of origin refuses to contribute to this particular public good.

Dr John William Devine (Adjunct Assistant Professor, Department of Philosophy, Trinity College Dublin) focussed on the moral mandate for flu vaccinations for health care providers (HCP). Maintaining high vaccination levels, which provides ‘herd immunity’, is an important public good: it is not so much my own immunity post‐vaccination that protects me from infection, as my being part of a vaccinated population in which infections are unlikely to be transmitted or take hold [13]. The key point here is that HCP themselves are unlikely to benefit from a flu vaccination as they are mostly young and healthy. Instead, the key beneficiaries of their vaccination are their vulnerable patients in whom vaccination may not generate sufficient immunity, and who rely on others not to pass the virus on.

The moral case in favour of vaccinating HCP is clear, and Devine listed three further reasons why HCP ought to get vaccinated: (1) The flu vaccine is not too invasive, very safe and inexpensive; (2) HCP have a special responsibility to promote their patients' health and a duty to do no harm; and (3) HCP might have an obligation to act as ‘role models’ and thus set an example with regard to immunization. Nevertheless, on average, less than 50% of HCP voluntarily consent to receiving the flu vaccine. How, Devine wondered, can this rate be increased? He explored three possible programmes: (1) mandatory vaccinations; (2) voluntary vaccination; and an (3) integrated programme for increasing compliance.

A mandatory programme would demand vaccination as part of the terms and conditions of employment. This would be inexpensive as no money would be spent on advertising, education or persuasion and it would be effective. Still, two main arguments count against it. First, it seems wrong to force people to receive injections. Second, it may affect our health care system for the worse by undermining its culture of trust and creating disaffected or resentful staff. We should not strive for *mere* compliance, Devine pointed out, but for *endorsed* compliance.

A voluntary programme fosters willing compliance by taking steps to persuade staff of the importance of the vaccine, and by making the vaccine easily obtainable. The advantage of this programme is that it avoids the problem of disaffected staff and interfering with autonomy, but its big drawback is that past experience suggests it is unlikely to result in high levels of compliance.

As a third option, Devine advocated for an integrated programme that has characteristics of both voluntary and mandatory programmes. The integrated programme draws upon education and persuasion to ensure that people mostly comply willingly, but also sees a role for mandatory vaccination where people do not comply willingly. This, Devine said, would increase vaccination rates without compromising autonomy (too much).

Much of the following discussion focused on Devine's endorsement of an integrated programme for compulsory vaccination of HCP. A central concern was that the strategy was not substantially different from enforced compliance and thus failed to respect autonomy – given that informed refusals ultimately would be rejected. The eventual enforcement might still lead to disaffection and distrust among health care staff, and might also make the attempt at persuasive techniques seem farcical and patronizing. In response, Devine argued that the aim of the integrated programme was to provide staff with the opportunity to reflect and voluntarily comply, reducing the amount of coercion involved overall.

In support of Devine's proposal, some argued that the integrated model struck the balance right: it would offer HCP the ability to ‘own’ their decision to receive the vaccine, thereby encouraging a higher level of compliance and perhaps even increased identification with and trust within the health care system. Another suggestion was that enforcement can have a positive role in persuasion: smoking bans were deeply unpopular when they first came into effect, precisely for reasons to do with autonomy. But now that they have been in place for a while, they are widely supported.

Some participants rejected the very idea that respect for autonomy should be such a central concern in the debate. While respect for autonomy is important, the compromise to autonomy within this policy is minimal; vaccinations would not be mandatory to all, but only a condition for getting a health care job. Many jobs have certain restrictions on what staff can do and require sacrifices. This is acceptable because people do not have a right to a (health care) job. In this context, it is important to remember that HCP are not just doctors and nurses; they also include, say, cleaners, catering staff, receptionists, technical staff, etc. While the former may have health‐related ‘professional obligations’, that is less clear in the case of the latter.

A further point of contention raised in discussion related to the evidence base for flu vaccines. Not everyone agreed that there was convincing rationale for such programmes, and HCP – they argued – have a professional obligation to criticize non‐evidence‐based policy, as well as a professional obligation to set a good example. Another point raised was that because the effectiveness of flu vaccines varies annually, and because the harms and benefits of individual (non) vaccination depends on baseline vaccination rates – which vary a lot between different health care groups – the force of coercion should at least vary with and be sensitive to these aspects.

Participants also discussed Greenhalgh's framework for considering health care problems in terms of conflicts between public goods and private interests. It was noticed that the notion of public good is ambiguous; Greenhalgh's definition is one used by economists, who are interested in scarcity, distribution and competition. But is that the best or even a suitable definition? What about goods that are not costly to oneself but that are simply enhanced by the activity of others? An example of this is singing in a choir. A wider conception of public goods may be necessary to accommodate, for example, the benefits of designing less lonely cities, or cycle lanes. In neither case does the concept of a ‘free rider’ make sense, for example, and both are important for physical and mental health. It was also suggested that thinking about private and public goods may be useful in thinking about health itself, which has characteristics of being both a private good – one's health is directly tied to one's own interest – and a public good – one's health affects other people's health in myriad ways, and other people's wallets through a nationalized health care and social security system.

Finally it was noted that Greenhalgh's example of the summary record was not just a case of public good versus private interest; as with the case of vaccinations, doctors may have had legitimate and non‐selfish concerns about the programme, such as its perceived threat to confidentiality and patients' privacy.

## Session 2: expertise and obligation

The session was opened by Dr Peter Freedman (Consultant Physician, Homerton University Hospital, London). Dr Freedman provided a comprehensive account of the historical use of the Hippocratic Oath. A central focus of the introduction was on how striking it is that reference is still made to a body of words formulated around 2400 years ago [14]. At the same time, Freedman pointed out, we should be wary of thinking that the Hippocratic Oath has been immutable or not open to interpretation: the Oath was co‐opted by the medical profession when Catholicism was dominant, for example, and at this point, its reference to pagan gods was omitted. The Oath has also undergone multiple translations [15, 16].

At present, the Hippocratic Oath is no longer (or at least not very widely) used formally, but its central tenets have more or less influenced many modern‐day codes of medical ethics. These central features are the duty to benefit the patient and to avoid harm, which are now instantiated in international codes of medical ethics [17, 18] and most recently in guidance from the General Medical Council (2013) [19]. Freedman raised the following points for discussion: whether taking an oath or a pledge is more effective than the imposition of a set of rules to which no explicit reference is made in an oath, and whether or not the Hippocratic Oath in either ancient or modern forms is still relevant.

Dr Emma Bullock (Postdoctoral Research Fellow in Concepts of Health, Philosophy Department, King's College London) argued that rather than rejecting the oath as outmoded, it must be reinterpreted in order to be applied in modern ethical codes. One way of understanding the duty of care (derived from the Hippocratic Oath) is that medical practitioners are obliged to protect and/or promote the well‐being of their patients through informed consent procedures. Specifically, she focused on claims made by Veatch [9] and Tännsjö [20] that complying with patients' free choice is the best means of discharging the duty of care since it protects the patient's well‐being as she conceives of it.

Bullock provided two objections to this particular reformulation: the existence of *epistemic handicaps* and the *maleficence* of promoting patient free choice. By the term *epistemic handicap*, Bullock meant that patients may not be in the best position to know what they want or what promotes their well‐being. In defence of this, Bullock cited evidence that patients suffer from cognitive biases and impairments – such as weakness of will, being subject to peer pressure and poorly judging the risk posed by a treatment option [21, 22, 23]. By *maleficence* of promoting choice, Bullock meant that the mere provision of information and/or choice can be detrimental to a patient's well‐being [24, 25]. For instance, the provision of genetic information can lead to stigmatization, psychological harm and discrimination [26]. The provision of choice can be discomforting and undesirable because patients may not want to choose, or in cases where the options available to the patent may be so unappealing that she lacks any preferences towards a particular treatment option at all.

Having rejected Veatch's reformulation of the duty of care as following patients' free choice, Bullock suggested two alternative options: first, a model that better balances patient autonomy with real concerns about the duty of care as highlighted above. This might include the provision of waiving informed consent when the provision of information is detrimental to patient well‐being. The second suggested alternative was a return to a paternalistic model of medical practice, based on the relative expertise of doctors, in which the patient is not always viewed as having decisional authority.

Subsequent discussion focused on four key areas, including (1) the practice and theory of informed consent; (2) the appropriate metaphor for the doctor–patient relationship; (3) the acceptability of a return to ‘paternalism’; and (4) the role of oaths.

First, many participants who were practising health care professionals readily affirmed that the theoretical ideal of informed consent and informed choice is exceedingly difficult, if not impossible, to enact in practice. In addition to familiar concerns about the impossibility of giving, let alone processing, all relevant information, real conflict could exist between acting in the patient's interest, respecting her rights and giving information. As an example, a practitioner reported that her patients sometimes expressed their wish that she had not given them certain information. This raises a conflict between the duty of giving information and the right not to know.

Second, there was discussion about what the right metaphor for doctor–patient relations ought to be. Some suggested that ‘maternalism’ might be better than paternalism because it is suggestive of a warmer, more empathetic and less authoritative or authoritarian relationship. Others strongly objected to such sexist metaphors: if anything, parentalism ought to be the term in use. It was specifically noted – with strong emotion – that the term ‘paternalism’ abused an outmoded, sexist model to derogate and dismiss attitudes of genuine concern for one's patient.

But many thought that the parent–child relation is an unsuitable metaphor for doctor–patient interaction in the first place because it is so unequal. Some suggested that a friendship model – a model that emphasizes equality, but also empathy and concern – would be a better metaphor [27]. Others thought friendship was not quite right since one ought not to befriend one's patients; they favoured an advocacy model.

Third, although it was widely acknowledged that informed choice was not fit for guaranteeing protection of the patient's best interests or discharging the doctor's duty of care, Bullock's proposal for relocating decision making with the health care professional met with mixed responses. Some pointed out that patients are already able to waive informed consent or ask doctors to make decisions for them – so no changes are needed here. Others pointed out that whether new or old, the waiving of informed consent is difficult to put into practice since the patient does not prospectively know what information might benefit or harm his/her interests.

While nobody objected to the idea that patients have epistemic handicaps, it was highlighted that health care professionals are suffering from epistemic handicaps, too; they often labour under pressures, such as exhaustion, insufficient time, targets and financial incentives. Nor should we assume that they are not influenced by emotions or false risk perceptions; health care professionals may be severely affected by and therefore overestimate the likelihood of the rare but atrocious harms they witness during their career.

Finally, it was pointed out that informed consent has more roles than ensuring patient's best interests; it has an important legal role in protecting doctors from charges of battery and assault. It also has an important role in protecting patients' autonomy rights to refuse physical interventions. Thus, the (much more minimal) requirement of informed consent as a means of protecting patients and doctors might need to be separated from the role of informed choice as a means of ensuring best outcomes. With respect to the latter, there was a definite preference for rebalancing autonomy with the duty of care under a nuanced model of trust and shared decision making.

A separate strand of discussion focussed on the role of medical oaths. On the one hand, it was queried whether such oaths are voluntary, given that they are necessary to work as a doctor, and whether taking an oath or adhering to a list of duties makes any discernible difference to medical practice. For instance, both Germany and Russia had statues for ethical medical research before the outbreak of WWII. On the other hand, it was noted that pledges have at least two important roles. First, taking an (outspoken) pledge to do something makes one more likely to do what is pledged; people who pledge not to give in to torture last longer under torture, and people who share their New Year's resolutions with others are more likely to stick to them. Second, oaths have a role in emphasizing and co‐opting a long cultural tradition, thus fostering cohesion, pride and trust in the medical profession.

At a deeper level, it was questioned why doctors, as opposed to other service providers (for instance, plumbers and bankers), have an oath. Does this follow from doctors being particularly unreliable – for, historically, oaths are often appealed to restore trust after it has been breached? Or is it because of the level of their expertise – the power and/or knowledge differential between doctor and patients? Or is it rather because of the seriousness or intimacy of the subject matter that we entrust them with? Although in comparison to plumbers' and doctors' expertise, we may be equally ignorant: plumbers at worst enter and flood our house; doctors may enter and kill our bodies.

## Session 3: evidence‐based medicine

The third session was introduced by Professor Brian Hurwitz (Professor of Medicine and the Arts, Department of English, King's College London). Hurwitz recounted his personal experience of creating and enacting the changing clinical practice of antibiotic treatment for acute infectious conjunctivitis (AIC) to then raise three topics for discussion: first, the role of clinical expertise in determining best practice; two, the role of research findings in determining clinical practice; and, three, the evolving nature of the ‘evidence base’.

AIC is an infection of the conjunctiva of the eye that is very painful, but often self‐limiting. In 20% of children and 50% of adults, the infection is of viral origin. Until 1995, the standard treatment for AIC was the application of broad‐spectrum antibiotics in the form of chloramphenicol drops. A *BMJ* editorial in 1995 advised against this in view of deaths arising from bone marrow aplasia directly related to the use of chloramphenicol [28, 29].

In light of this publication, Hurwitz undertook meta‐analyses of heterogeneous randomized trials looking at the effectiveness of antibiotics for AIC. Both the first and second meta‐analysis found AIC to be self‐limiting, with no significant symptomatic benefits of antibiotics; 65% of symptoms were cleared on days 2–5 with the use of a placebo [30, 31, 32]. While topical antibiotics were found to be of some benefit in improving early clinical and microbiological remission, these benefits were significantly reduced in later stages. In light of these findings, and ‘following the evidence’, Hurwitz changed his clinical practice and no longer prescribed chloramphenicol for conjunctivitis.

However, upon discovering that his patients were exceedingly distressed by this change and by their unrelieved painful symptoms – which, as Hurwitz' clinical observation, had seemed to clear up quicker under antibiotics – Hurwitz reluctantly reversed his practice back to prescribing chloramphenicol, thus following his clinical observation and going ‘against the evidence’.

A later and bigger meta‐analysis vindicated Hurwitz observation: although there was no difference between antibiotics and non‐antibiotics in outcome measures at day 3, in the treated group, symptoms did resolve themselves quicker, thus explaining his observation of quicker symptom relief in his antibiotics‐treated patients [33].

On this basis, Hurwitz advocated a nuanced relationship between evidence and practice, with a considerable role for clinical judgment and expertise, and flexibility to determine the right course of action in light of the patient's circumstances; for some, a few hours difference in onset of symptom relief might make a world of difference, but for others, practically none. He also suggested that while the ‘the evidence’ is sometimes definitive, it is also often evolving and may not capture everything of note: it failed to capture the notion of ‘onset of symptom relief’ and, in this instance, it seemed the evidence had ‘lagged behind’ clinical judgement.

Hurwitz's discussion was followed by Dr Luis José Flores (Department of Philosophy, King's College London, and Department of Psychiatry, Pontifical Catholic University of Chile), who examined problems for the reasoning processes involved in justifying the ‘hierarchy of evidence’ and in applying that hierarchy to individual cases.

Evidence‐based medicine's so‐called ‘Hierarchies of Evidence’ privilege evidence from randomized controlled trials (RCTs) and in particular meta‐analyses (MAs) of RCTs above all other sources of knowledge. Applying such knowledge to individual cases, Flores pointed out, relies on the following inferential syllogism:

**1** 
Meta‐analyses/best evidence show that most cases of diagnosis *D* respond to treatment *T*.
**2** 
It is likely that the patient has *D*.
**3** 
This is all I know about the matter.
**C** 
Therefore, it is reasonable to conclude that the patient will respond to intervention *T*.

Using this syllogism, Flores argued, we can first see that the strength of the conclusion depends on the certainty of premise 1, which is dependent both on the strength and certainty of the research findings, as well as the amount of trust we put into the evidential sources. It is with respect to this premise that EBM, through the use of its hierarchies, attempts to increase the strength and reliability of the evidence base. But that is not all that determines the strength of the conclusion.

The above inference, Flores pointed out, also involves an inference from a group probability in (1) to an individual probability (C). This, Flores, argued, is the problem of generalizability of research findings, which has two aspects. First, this involves a generalization of research findings from the research population to the treatment population. These may not be identical; research populations are often younger, more homogenous, more typical in their presentation and with lower levels of co‐morbidity than the treatment populations. Second, it involves an inference from a group probability to an individual probability. This is not an inference that has much warrant: a group displaying a certain correlation can have very many members to whom this correlation does not apply, or applies in reverse.

Such an inference is only warranted if it is the very best we can do – if premise (3) is true and this is all we know about the matter. But premise (3) is nearly always false: we know much more about the matter at hand, and, in particular, we know details about our individual patients.

Having explained thus far, Flores proposed two possible ways in which EBM could improve on dealing with the generalizability problem. The first is to make the research population more like the target population/individual through, for example, pragmatic trials, *n* = 1 trials and a refinement of reference classes. This solution would improve things, but it could not get rid of generalizability problems. The second is to reject the idea, implicit in the above syllogism that meta‐analyses are our only and therefore ‘best guess’, and instead combine this knowledge with other knowledge that we possess, including, for example, clinical experience, data mining, subgroup analysis and pathophysiological reasoning in order to arrive at a best guess based on a combined knowledge source.

In the subsequent discussion, there was widespread agreement that there is an intractable, insoluble problem of applying population data to individual patients; evidence from RCTs gives probability, but no amount of RCT‐based evidence can close the inferential gap between population and individual. Further problems for a pure reliance of RCTs were noted: we do not need an RCT to know that we need to act on obstructed tracheas and in the case of other dramatic results, and RCTs are easily corrupted by the powers that finance them through choice of outcome measures, publication bias and other mechanisms that are well documented in the literature [34].

A further theme in the discussion was the role of values in individual treatment and in research; treating a patient must be guided by what the patient wants, and that may not be what the HCP think the patient wants, or what the researchers thought patients would want. This linked in with two other points. First, what looks like factual evidence is not chosen in a value‐free way: the outcomes of RCTs are fixed in factual terms, but how these outcomes are formulated reflects the perceived desirability of different outcomes. Thus, the research in Hurwitz' example focussed on symptom resolution after 3 days, not on what was the patient‐relative outcome: the time‐to‐onset of noticeable pain relief. Had the research taken that, or symptoms at 12 and 24 hours as an outcome, it was suggested, Hurwitz might well have found a discernible difference in his first meta‐analysis. Similarly, it was noted, claims about the ineffectiveness of antibiotics for other common conditions such as otitis media and sore throat often refer to symptom resolution after 5 days. But for children, and their parents, what matters is the duration of the really painful bit – and there even a couple of hours can make a huge difference, although in a research paper that is unlikely to be taken as relevant. Similarly, treatment outcomes often focus on ‘objective measures’, such as a photo or a blood test, which may not correlate with the outcome that matters for the patients. A second point was that RCTs focus on large numbers and can therefore focus on generating results that can be made statistically significant. But, participants pointed out, these results may not be clinically significant: they may not matter for individual people. This tied into a larger worry about RCTs: that in forcing a particular framing of questions – a focus on manipulable, single causes – it closes the door on investigating important relationships that cannot so easily be fit into that model, for example, long‐term exposures to diffuse social contexts.

There were also more critical voices of these lines of thought. Some thought that in devising guidelines, factors were taken into account – such as the development of antibiotics resistance – which individual patients were unlikely to consider, but that, in a nice link back to the first topic of the day, should be considered.

With respect to clinical experience and Hurwitz's case study, a lively suggestion ensued. Some suggested that Hurwitz should not have resorted to antibiotics, but simply to eye drops, which can have a washing and pain‐relieving effect. Since antibiotics are administered as drops, part of their effect may have been due to this. Placebo‐controlled studies will have compared antibiotics not with *no treatment* – which is what Hurwitz proceeded to offer his patients – but with *eye drops without AB*. Another explanation, offered by a long‐practising GP, for the changing evidence was that people have become a lot better at diagnosing conjunctivitis during her lifetime: not every sticky eye is conjunctivitis anymore. At the same time, it was thought that clinical experience is better at picking up small effect sizes than is commonly acknowledged.

## Session 4: person‐centred medicine, particularism and judgement

The final session opened with an introduction from Dr James Appleyard (retired consultant paediatrician). Appleyard suggested that the view that patients are persons is not mainstream. We should consider patients as persons primarily because a patient is limited in descriptiveness and implies lack of autonomy and dependency; ‘patient’ characterizes a patient primarily as a sufferer, which emphasizes only one aspect of the person. Historically, doctors have been patronizing to patients identifying them with terms such as service user, client, consumer [35, 36, 37].

Appleyard adopted a four‐part definition of person‐centred medicine: (1) medicine *of* the person (of the totality of the person's health including its ill and positive aspects); (2) medicine *for* the person (promoting the fulfilment of the person's life project); (3) medicine *by* the person (with clinicians extending themselves as full human beings well‐grounded in science and high ethical aspirations) and (4) medicine *with* the person (working respectfully in collaboration and in an empowering manner through a partnership of patients, family and clinicians) [38].

Treating a patient as a person, Appleyard maintains, prevents HCP from treating disease primarily. This is important because the social determinants of disease are overwhelming; if social factors are not taken into account, treatment outcomes will not be satisfactory. For example, research on the frequency of throat infections in children shows that there is a big correlation between events in family and incidence of throat infections [39, 40].

Person‐centred medicine also involves the recognition that a doctor is a person, too, with certain attributes, skills and values. Ideally, doctors would be matched with the patient in terms of each of their personal characteristics. Understanding the doctor as a person facilitates a proper dialogical relationship with patient as a person fostering connectedness and thereby enhancing patient well‐being.

Appleyard noted that the person‐centred approach to medicine is often thought to be associated with increased rather than decreased resource utilization. However, studies from the University of Gothenburg and others show decreased utilization and increasing patient satisfaction both in acute and chronic conditions on the person‐centred model. For instance, it was reported in a study of patients admitted to hospital with hip fracture that there were fewer medical complications and a 40% reduction in average total cost with no readmissions [41]. Person‐centred care fosters a feeling of connectedness with an interpersonal outlook of unity which promotes attitudes of hope, empathy and respect. With the enhancement of well‐being, drop out relapse and recurrence rates in physical and mental disorders are reduced [42, 43].

Professor Jonathan Wolff (Professor of Philosophy, University College London) explored the philosophical doctrine of moral particularism as a route to developing an account of medical particularism. We are inclined to think, Wolff argued, that the purpose of medicine is to cure disease, and that the reason we visit doctors is to obtain a cure. But if that is so, he said, we are sorely disappointed. Medicine very often does not have a cure, in fact until quite recently, medicine pretty much failed to cure anything at all [44, 45, 46]. Since the discovery of surgery and antibiotics, medicine sometimes cures things, but even then our powers of diagnosis far outstrip our curative powers [47].

Why is curing so difficult? One, common sense, answer is to think that we simply do not know enough (yet). On this picture, there could in principle be a *manual* to the human body, just as there is a manual to your car. With this manual, describing the full set of rules for how the body functions and dysfunctions, in hand, we would understand everything about the human body, and could cure it. But we just do not have (and may never be able to obtain) that manual. In principle though, on this picture, the manual could exist; thus our failure to cure is an *epistemic failure*.

There is, however, another way to think about the human body, Wolff suggested. This idea, medical particularism, is inspired by the approach to philosophical ethics called ‘moral particularism’. Moral particularism maintains that we cannot give a full set of rules that accurately describe, or from which we can deduce, the moral facts in every situation. Thus, a moral judge needs far more than a set of rules or principles to proceed. These, at best, serve as a set of crutches or a stepping stone, but would lead to moral error if universally and rigidly applied [48].

Similarly, a *medical particularist* would not assume that everything fits neatly together – that there is or could be a *manual* for the human body – and will have to proceed in absence of such a rulebook. Thus, a medical judge, too, would need far more than a range of medical principles, rules, regularities or research findings and the ability to apply them to be a good medical judge. If medical particularism is a plausible approach to medicine, then that gives a reason to reject the simple or rigid application of evidence‐based rules of guidelines, and may give a justification for person‐centred medicine.

In the following discussion, there was a notable enthusiasm for the move towards person‐centred medicine and away from the increasing depersonalization of patients to biological entities. It was speculated that person‐centred medicine has the potential to achieve better diagnoses. For instance, if one were to consider in the context of the patient as a person whether a test was needed (as opposed to relying on EBM), HCP are likely to give a more timely diagnosis to their patients.

Two central concerns with person‐centred medicine and medical particularism were discussed in detail. First, there was some apprehension that personalized and particularist approaches to medicine presuppose a careful ongoing relationship between the patient and doctor. It was noted that the success of moral particularism depends on having an attuned moral sensitivity that can only be developed over time. However, the ability to develop an analogous ‘medical’ sensitivity towards patients does not fit with the current realities of health care and primary care management, which are constrained in terms of time and finance.

Participants were also concerned with the complexity of medical particularism. While it was agreed that this approach correctly identifies the inadequacies of reductionist or generalist approaches to medicine, it remains unclear how all of the particular features of an individual patient can be taken into consideration in the medical setting. A possible solution to this was suggested in the form of a requirement that HCP work within a supportive network and have a significant amount of information about the particular patient they are treating. It was disputed as to whether this solution was itself practically feasible.

In light of the current socio‐economic difficulties of fostering long‐term relationships between HCP and patients, the discussion concluded with the suggestion that general principles are in fact needed. However, while being necessary, it was agreed that general medical principles should not be treated as sufficient for correct medical practice and should play more of a subservient role than they currently do. For this reason, person‐centred medicine was recognized as an attractive alternative, allowing HCP to apply informed general theories in a way that is sensitive to each patient's particular needs.

## Conclusions

As with the previous four workshops in the series, the fifth CHH interdisciplinary philosophy and medicine workshop was a huge success. Throughout the day, there was a balanced discussion between clinicians and philosophers, unhindered by terminological confusion; only one request for terminological clarification was raised. The central outcome of the day was a significant support for a move towards more person‐centric medicine. This was apparent in discussions relating to both of the two key areas of the workshop: medical knowledge and medical duties.

Under the heading of ‘Medical Knowledge’, it was widely agreed that general principles are inadequate for providing appropriate patient care. Session 3 emphasized the failures of EBM to identify the best (or, the least worst) medical treatment for individual patients. First, the aims of RCTs were thought to be at odds with what matters for the patient, who generally prefer to know how long their symptoms will be painful as opposed to how long their overall condition will last. Second, it was noted that population studies cannot address the particular needs of the individual. While this problem was thought to be intractable, it was also believed that an appropriate solution was to give more control to the clinician's judgement. The level of clinical judgement needed was expanded upon in discussions in session 4 with the model of person‐centred medicine. Here, it was argued that clinical judgement can be better informed by treating patients as individual persons. It was speculated that as a result of adopting this model, HCP will reach more accurate diagnoses and deliver better treatment.

The second central area of discussion on ‘Medical Knowledge’ related to controversies over the credibility of research backing flu vaccination schemes. Discussion in session 1 emphasized the need for HCP to use their clinical judgement, but this time in terms of the obligations they have towards themselves; HCP should have the freedom to question the validity of research backing vaccination schemes, such as whether the research is biased, before committing to the schemes.

This focus on the importance of clinical judgement bore a direct relationship to discussions on the professional *duties* that HCP have towards themselves and the ethical *duties* that they have towards their patients. In the context of the discussion on mandatory vaccination programmes, it was argued that a degree of HCP autonomy remained important. Some even suggested that HCP are *obliged* to criticize policies with an inadequate evidence base. With respect to patients and the HCP's ethical duties, it was agreed that a real conflict exists between respecting patient autonomy and acting under the duty of care, with the ideal of informed consent being impossible to enact in practice. It was suggested that there is a need to rebalance autonomy with the duty of care under a model of trust and shared decision making.

Overall, the workshop provided significant support for a movement towards more nuanced models of applying and gaining medical knowledge in both medical research and therapeutic contexts. On the basis of the difficulties of implementing these more complex models, and the duties they entail, a further workshop was planned on ‘Parentalism and Trust’ in medicine (June, 2014). This workshop would provide more focus on the ethical and epistemic privileges of clinical judgement and the clinician's relationship with the patient. The title of the workshop, ‘Parentalism and Trust’, was chosen to reflect concern arising during the workshop that the term *paternalism* has unfortunate sexist and authoritative connotations. It was advised that the workshop on ‘Parentalism’ should address the persistent concern of the day: that in order for clinical judgement to successfully buffer the inadequacies of EBM, and for person‐centred medicine to achieve good treatment outcomes, a trusting relationship between the HCP and the patient is needed.

## Footnotes

1
http://www.kcl.ac.uk/innovation/groups/chh/. Funded by a Wellcome Trust Strategic Award (Number 086071).
